# Suppressing Self-Discharge with Polymeric Sulfur in Li-S Batteries

**DOI:** 10.3390/ma12010064

**Published:** 2018-12-25

**Authors:** Min Jiang, Bingqing Gan, Yongqi Deng, Yin Xiong, Ruixuan Tan

**Affiliations:** 1 School of Materials Science and Engineering, Shanghai Jiao Tong University, Shanghai 200240, China; jiangminhh@sjtu.edu.cn; 2 College of Materials Science and Engineering, Changsha University of Science & Technology, Changsha 410114, China; bingqinggan@126.com (B.G.); dengyongqi25@126.com (Y.D.); xy1604734707@126.com (Y.X.); 3 Science and Technology on Advanced Ceramic Fibers and Composites Laboratory, College of Aerospace Science and Engineering, National University of Defense Technology, Changsha 410073, China

**Keywords:** polymeric sulfur, lithium-sulfur battery, shuttle effect, self-discharge

## Abstract

Lithium–sulfur (Li-S) batteries, due to their high theoretical capacity, intrinsic overcharge protection, and low cost, are considered as the most promising candidates for next-generation energy storage systems. To promote widespread use of Li-S batteries, various tactics have been reported to improve the columbic efficiency and to suppress the shuttle effect. Herein, we report a novel polymeric sulfur via heat radical polymerization, for the Li-S battery. The insolubles after CS_2_ washing, and the changes in XRD (X-ray diffraction) results imply the formation of polymeric sulfur. Owing to the absence of cyclic S_8_ molecular, the shuttle effect is depressed, and the polymeric sulfur cathodes exhibit lower self-discharge rates, higher sulfur utilization, better rates of performance, and enhanced cycling stabilities than the commercial sublimed sulfur. Thus, polymeric sulfur provides a new train of thought and tactics for restricting the formation of the dissolution of polysulfides, and self-discharge.

## 1. Introduction

The lithium–sulfur (Li-S) battery is one of the most promising candidates in rechargeable batteries, due to their high energy density [1]. Compared with conventional lithium insertion hosts, such as the olivine LiFePO_4_ and layered LiCoO_2_, sulfur is an abundant and environmentally friendly material, and it has an especially high theoretical energy density (over 2600 Wh/kg) [2]. However, the practical applications of the Li-S batteries are impeded by several major issues, due to the insulating character of sulfur, large volume expansion (form S to Li_2_S) and the shuttle effect during the redox process [3]. The generated polysulfide ions can be dissolved and transferred through the electrolyte, and react with the lithium anode, resulting in a low columbic efficiency and rapid capacity fading [4]. Another challenge in Li-S battery development is severe self-discharge [5], which means that the battery will lose its charge–discharge capacity without any connection between the electrodes when stored for a long time. The phenomenon of self-discharge depends on many factors, such as the electrode composition, current collection, and the storage temperature. Beyond that, it is certain that the existence of polysulfide ions in the electrolyte for Li-S batteries has been recognized as one of the key factors for shuttle effect and severe self-discharge. Mikhaylik and Akridge reported on the quantitative analysis of the shuttle phenomenon [6]. It was reported that the self-discharge is primarily due to the high plateau of polysulfides (Li_2_S_x_, 8 < x < 3).

In order to address these issues, many approaches were adopted, including a modified sulfur host, electrolyte additives, and protection of the anode [7]. A major current focus is the controlled synthesis of functional nanostructures of sulfur and carbon composites for suppressing the shuttle phenomenon, and then improving its cyclability and conductivity [8,9,10]. Furthermore, with the development of lithium anodes, some scientists pay more attention to anode material, such as the protection of lithium using artificial SEI [11]. However, little attention has been given to the direct processing of elemental sulfur without any host materials. According sulfur physical properties, there exist various speciations of sulfur with phase and temperature. Depending on the different environments, the chemical behavior will vary [12]. As is well-known, cyclic sulfur (α-S_8_) is the most stable allotrope at room temperature, and it undergoes a series of structural and morphological changes during the discharge process involving the formation of soluble lithium polysulfides Li_2_S_x_ (3 < x < 8), causing the so-called shuttle phenomenon [13]. Guo et al. [14] demonstrated that the S_2-4_ rather than cyclic S_8_ can skip the step involving the soluble lithium polysulfides, due to the absence of the high plateau at about 2.3 V ($S_{8}\to S_{4}^{2-}$) [15]. However, it is impossible to guarantee sulfur uptake for small non-cyclic allotropes, causing the energy density target to be difficult to achieve. We need to know whether it is possible to use linear polymeric sulfurs for Li-S batteries. Research shows that the low barrier for the ring-opening reaction of cyclic sulfur renders the element capable of forming linear chains, due to the energy requirement being only 36 kcal/mol [12]. Li-S batteries with synthetic polymeric sulfur can skip the step involving the soluble lithium polysulfides during the first discharge, and significantly reduce the efficiency of self-discharge when stored. This is because the unfavorable transition from S_8_ to $S_{4}^{2-}$ is avoided and the soluble lithium polysulfides are consequently prevented from discharging.

In this work, we reported on the self-discharge characteristics of a Li-S battery with polymeric sulfur. The polymeric sulfur was synthesized by the processes of free radicals via heat treatment. The results suggested that the generated polymeric sulfur can effectively suppress self-discharge during the redox process.

## 2. Materials and Methods

Materials: Sublimed sulfur (Sigma-Aldrich, Shanghai, China), H_2_O_2_ solution (10%, Sigma-Aldrich), Carbon disulfide (CS_2_, Sigma-Aldrich). All reagents were purchased from commercial sources and used without further purification.

### 2.1. Preparation of Polymeric Sulfur

In a typical procedure, 2.0 g sulfur powder was sealed into a glass tube. Subsequently, the glass tube with the sublimed sulfur was arranged in a thermostatic oil bath (DU-30G, Yiheng, Shanghai, China). The sulfur powder was heated to different temperatures (160 °C, 200 °C, 240 °C, 280 °C, 320 °C) with various treatment times (2 min, 3 min, 4 min, 5 min) to obtain polymeric sulfur. After the heat treatment, the glass tube was immediately immersed in a 10 wt % H_2_O_2_ aqueous solution for 5 min to remove free radicals of sulfur. The obtained precipitate was collected by filtration, washed with methanol several times, and then soaked with CS_2_ for 30 min to remove the soluble sulfur. The insoluble precipitate was dried at 50 °C for 12 hr, and the polymeric sulfur was obtained.

The yield of polymeric sulfur was calculated by the difference of the precipitate’s weight before heat treatment and after washing with CS_2_.

### 2.2. Materials Characterization

The X-ray diffraction (XRD, Ultima IV, Rigaku, Shanghai, China) spectra of the polymeric sulfur were characterized using a Philips X-ray diffractometer with Cu K_α_ radiation (*λ* = 1.54187 Å) at a speed of 2°/min. The morphology of the polymeric sulfur was measured by using scanning electron microscopy (SEM, S-4800, Hitachi, Shanghai, China).

### 2.3. Electrochemical Characterization

Cathodes were prepared by casting the N-methyl-2-pyrrolidone slurry containing 80 wt % polymeric sulfur, 10 wt % conductive carbon (super P), and 10 wt % PVDF (polyvinylidene fluoride) on the aluminum foil. The loading of sulfur was around 2.6 mg/cm^2^. The electrochemical behavior was tested by assembled CR2012 coin cells in a glove box (LABstar, MBRAUN, Shanghai, China) by employing the polymeric composites as cathode, the microporous polypropylene membrane (Celgard 2400) as the separator, and lithium foil as the counter electrode. The electrolyte is 1 M lithium bis(trifluoromethanesulfonyl)imide in a mixture of 1,2-dimethoxyethane and 1,3-dioxolane 1:1 (v/v) solution. Cyclic voltammetry curves were carried out by using a CHI 660E electrochemical workstation (Chinstruments, Shanghai, China) at a scan rate of 0.1 mV/s from 3.0 to 1.4 V. The electrochemical cycling tests of the cells were galvanostatically performed between 1.4 and 3.0 V (versus Li^+^/Li) at various charge/discharge current densities.

## 3. Results and Discussion

### 3.1. Physical Characterizations

**Figure 1:**
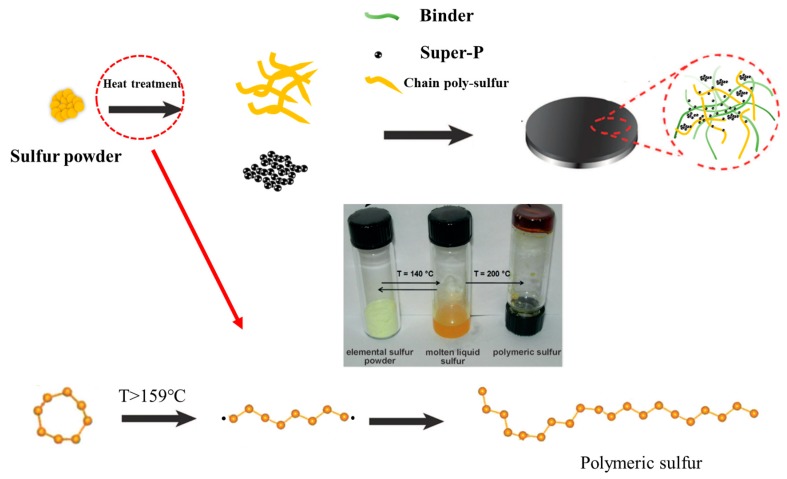
Design and preparation of a chain poly-sulfur/Super-P composite integrated cathode.

A typical synthesis route of polymeric sulfur is schematically depicted in Figure 1. First, the glass tube with sublimed sulfur was immersed into a constant temperature oil bath to heat the commercial sublimed sulfur. As is well known, the energy barrier for the ring-opening reaction of cyclic S_8_ is only 36 kcal/mol. During the heat treatment, the eight-membered ring structure was destroyed as the ring-opening polymerization reaction occurred, and the chain of polymeric sulfur grew. With the rapid cooling by the H_2_O_2_ aqueous solution, the thermal radical reaction was terminated, and the closed end of the polymeric sulfur was stabilized by the H_2_O_2_. The polymeric sulfur was purified by CS_2_ washing, and then prepared as a cathode, with super P as the conductive carbon and PVDF as binder. The electrochemical performance was performed in the coin cell.

**Figure 2:**
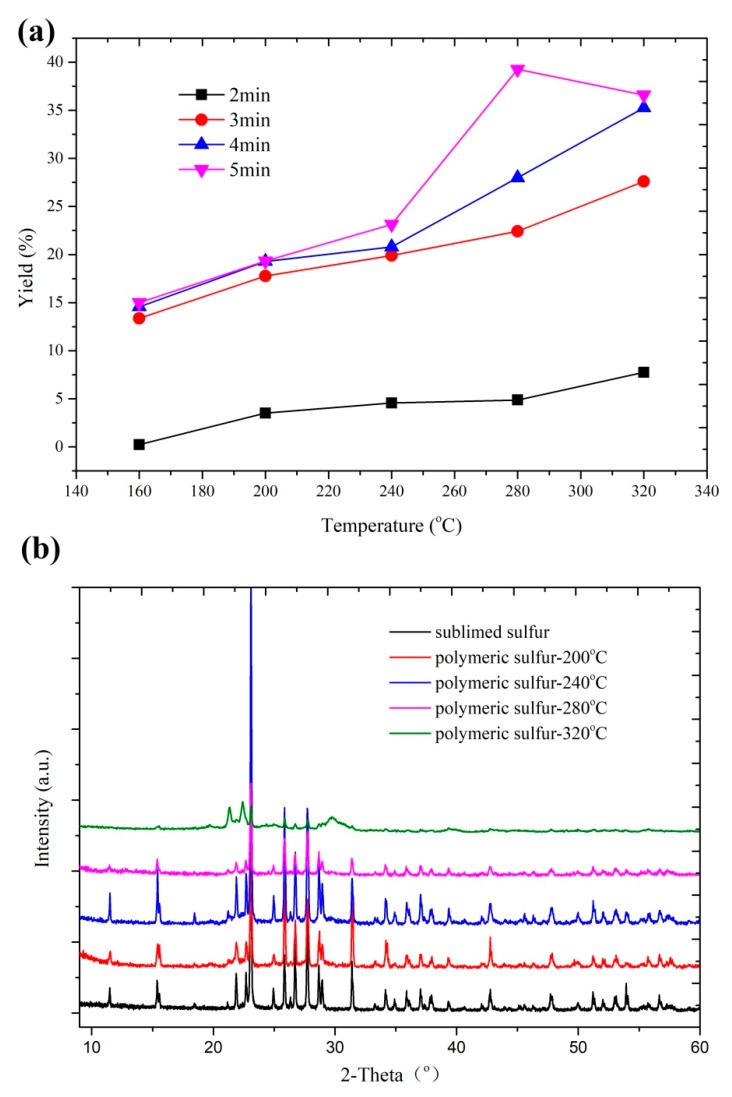
(**a**) Yield of the polymeric sulfur at different temperatures and reaction times. (**b**) XRD patterns of the commercial sublimed sulfur and the polymeric sulfur prepared at various temperatures for 4 min.

Thermal treatment temperature and time have important influences on the yield of the polymeric sulfur. Figure 2a represents the yield of the polymeric sulfur as a function of reaction temperature and time. It is shown that the thermal treatment maintains 2 min, and a low yield of polymeric sulfur is obtained. As the time extends to more than 3 min, the yield is greatly improved. The yields of the polymeric sulfur increase with the increase of temperature, and the highest yield is 39.26%, at 280 °C for 5 min. The positive correlation between the yield of polymeric sulfur and the thermal treatment temperature and time was due to the endothermic ring-opening reaction [16]. However, the small molecular (S_2-4_) would generate and evaporate, which caused a low yield of polyermic sulfur once the thermal treatment temperature was set to as high as 320 °C for 5 min. Figure 2b shows the XRD patterns of the commercial sublimed sulfur and the polymeric sulfur obtained at different temperatures. The XRD patterns of the pristine sulfur at 15.5°, 22.5°, 23.1°, 25.9°, 27.8°, and 31.4° could be well indexed into standard sulfur with JCPDS card no. 08-0247. The diffraction peaks located at about 23.1°, 25.9°, and 27.8° were attributed to the (222), (026) and (040) diffractions of orthorhombic structure of crystalline sulfur. With the rise in temperature, the peak intensities of these characteristic peaks were gradually weakened. When the temperature rose to 320 °C, most of the diffraction peaks almost disappeared, and the diffraction peaks located at about 21.3° and 22.5° shifted left, meanwhile, with the appearance of a new broad peak at approximately 29°. These results indicate that α-sulfur structure of commercial sublimed sulfur transforms to a new structure.

**Figure 3:**
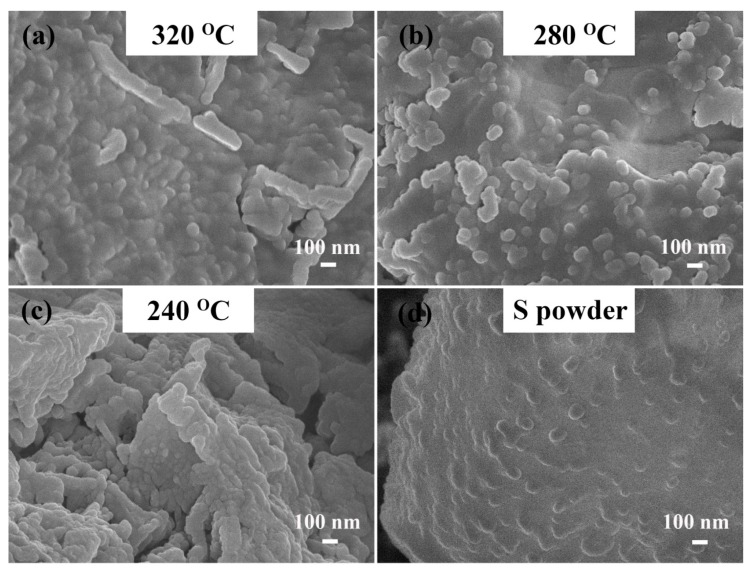
SEM images of commercial sublimed sulfur power and the polymeric sulfur prepared at different temperatures for 4 min (**a**) 320 °C; (**b**) 280 °C; (**c**) 240 °C; (**d**) room temperature.

Figure 3 displays the SEM images of the as-prepared polymeric sulfur at different temperatures (a–c), and the commercial sublimed sulfur powder (d). The polymeric sulfur presented a rough surface, and it was agglomerated with a larger number of smaller particles than that of the commercial sublimed sulfur. These coarse morphologies ensured a larger specific surface area, which would be conducive to electrochemical performance.

### 3.2. Electrochemical Characterizations

**Figure 4:**
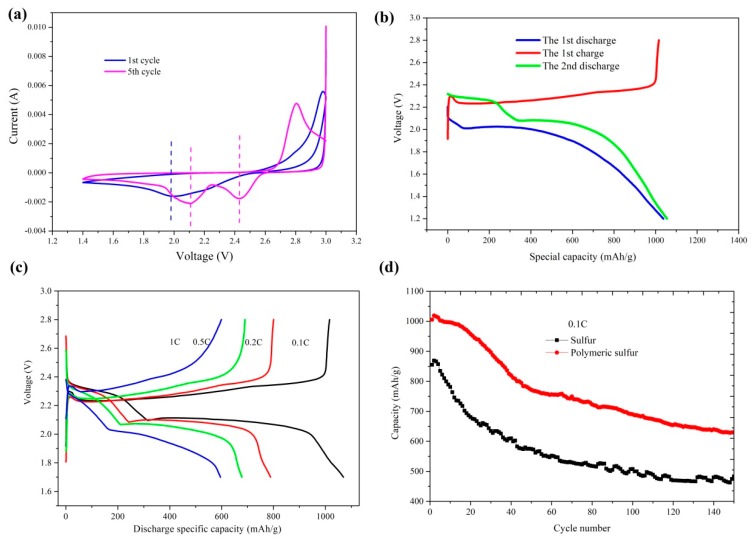
(**a**) CV curves of the polymeric sulfur in the first and fifth cycle between 1.4 V and 3.0 V with the sweep rate of 0.1 mV/s. (**b**) Discharge performance of the commercial sublimed sulfur and the prepared polymeric sulfur in the first and fifth cycles. (**c**) Charge–discharge performance of the prepared polymeric sulfur at different rates. (**d**) Long-term cycle performance of the sublimed sulfur and the prepared polymeric sulfur at 0.1 C.

The cyclic voltammetry (CV) test was performed to understand electro-chemical behavior (Figure 4a). It is notable that the catholic peaks appeared at around 2.05 V and 2.3 V for the polymeric sulfur, corresponding to the discharge plateau of PS. Only one pair of redox peaks was observed in the CV curve at the first cycle, while one oxidation peak at 2.8 V and two reduction peaks at about 2.05 V and 2.3 V were presented at the fifth cycle. The absence of the plateau at about 2.3 V (S_8_$\to S_{4}^{2-}$) in the first CV cycle indicated the absence of cyclic S_8_, as well as the polysulfide that caused the shuttle effect. With increased cycling, the small S_2-4_ molecules began to generate the common cyclo-S_8_. After a few cycles, the fifth CV curve was similar to that of the commercial sublimed sulfur, which might have been attributable to the formation of cyclic S_8_ during the charge process. As is known, the self-discharge of the commercial Li-S batteries is very serious under real operation, due to the shuttle effect. Thus, the polymeric sulfur presents significant self-discharge inhibition performance during the battery storage.

Figure 4b demonstrated the charge–discharge curves during first cycle and the second discharge curves of the polymeric sulfur at 0.1 C. During the first discharge, the discharge curve showed only one voltage plateau at approximate 2.0 V, while two voltage plateaus at 2.1 V and 2.3 V were present during the second discharge process. This confirms that during the first charge process, cyclic S_8_ was formed. The 2.3 V discharge plateau was slightly lower than the reduction peak in the fifth CV curve in Figure 4a, indicating the incomplete conversion of polymeric sulfur to cyclic S_8_.

Figure 4c shows the charge–discharge performance of polymeric sulfur at different rates. Discharge capacities of 1069, 788, 679, and 595 mAh/g were delivered at various rates of 0.1 C, 0.2 C, 0.5 C, and 1 C respectively. The general rate performance was probably due to the poor conductivity of the polymeric sulfur.

Figure 4d compares the long-term cyclability of the synthesized polymeric sulfur and the commercial sublimed sulfur at 0.1 C. In the first few cycles, the capacity of polymeric sulfur increased a little, and then gradually decreased as the cycle number increased. This may be caused by the incomplete consumption of polymeric sulfur during the beginning cycles [17].

**Figure 5:**
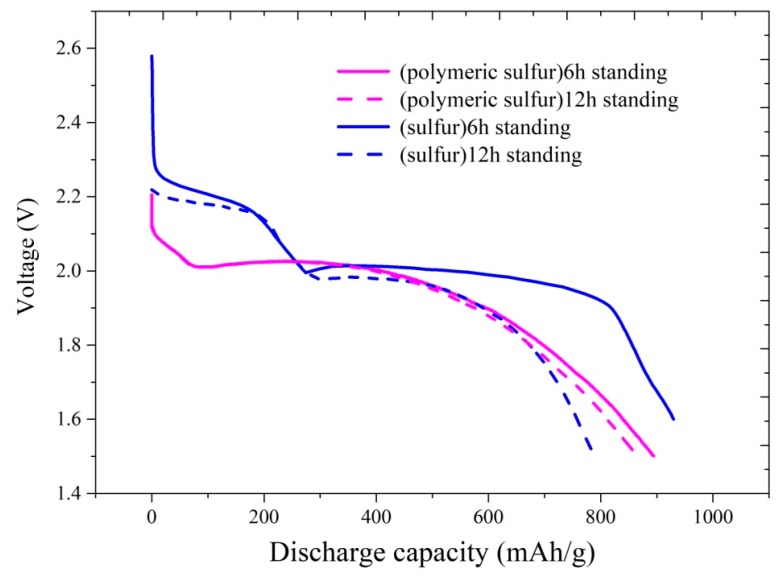
Discharge curves of the Li-S cells with commercial sulfur and the synthesized polymeric sulfur as a cathode after resting for 6 h and 12 h.

In order to further investigate the self-discharge behavior of the cells with different cathodes, the discharge curves of the cell were compared between the commercial sulfur and the polymeric sulfur cathode. As shown in Figure 5, the self-discharge capacity loss of the cell with various standing time was lower than the commercial sulfur.

## 4. Discussion

According to the discharge performance of the polymeric sulfur after resting, both the cell voltage and the specific capacity of the cell with commercial sulfur were dropped sharply after rested for 6 h and 12 h. The voltage drop is likely due to the shuttle effect, which is caused by the dissolution of the cyclic S_8_ in the electrolyte, and then reduced in the anode side. However, due to the absence of the S_8_, the cell with polymeric sulfur cathode shows almost no change on the cell voltage after resting. This indicates that the employ of the polymeric sulfur as the cathode of Li-S battery would presents a significant self-discharge inhibition performance. The reversible storage capacity of the Li-S cell is irreparably diminished. Thus, it is very meaningful for us to store the Li-S cell via the polymeric sulfur. Additionally, the polymeric sulfur shows better performance during the whole cycle life and the capacity retention is 58% after 150 cycles, while that for commercial sublimed sulfur is only 54%. These results suggest that the polymeric sulfur is a positive cathode material for Li-S battery, which is more promising than that of sublimed S_8_ sulfur.

## 5. Conclusions

In this study, the polymeric sulfur is synthesized via heat radial polymerization, and it was tested in Li-S batteries. A max polymeric sulfur yield of 39.26% was achieved at 280 °C for 5 min. The polymeric sulfur exhibited a good effect on depressing the shuttle effect in the Li-S battery, due to the absence of cyclic S_8_. The polymeric sulfur presents a better cycle performance than that of the commercial sublimed sulfur. Additionally, the polymeric sulfur presents significant self-discharge inhibition performance during battery storage. Unfortunately, the polymeric sulfur would partly transform to cyclic S_8_ during the charge–discharge process. Also, for the consideration of better cell performance, the conductivity needs to be further improved. This novel polymeric sulfur provides new ideas for the development of lithium–sulfur batteries.
